# Whole exome sequencing of familial hypercholesterolaemia patients negative for *LDLR*/*APOB*/*PCSK9* mutations

**DOI:** 10.1136/jmedgenet-2014-102405

**Published:** 2014-08

**Authors:** Marta Futema, Vincent Plagnol, KaWah Li, Ros A Whittall, H Andrew W Neil, Mary Seed, Stefano Bertolini, Sebastiano Calandra, Olivier S Descamps, Colin A Graham, Robert A Hegele, Fredrik Karpe, Ronen Durst, Eran Leitersdorf, Nicholas Lench, Devaki R Nair, Handrean Soran, Frank M Van Bockxmeer, UK10K Consortium, Steve E Humphries

**Affiliations:** 1 British Heart Foundation Laboratories, Centre for Cardiovascular Genetics, Institute of Cardiovascular Science, the Rayne Building University College London, London, UK; 2 Department of Genetics, Environment and Evolution, UCL Genetics Institute, University College London, London, UK; 3 Department of Primary Care Health Sciences, NIHR School of Primary Care Research, University of Oxford, Oxford, UK; 4 Department of Cardiology, Imperial College Health Services, Charing Cross Hospital, London, UK; 5 Department of Internal Medicine, University of Genoa, Genoa, Italy; 6 Department of Biomedical, Metabolic and Neural Sciences, University of Modena and Reggio Emilia, Modena, Italy; 7 Centre de Recherche Médicale de Jolimont, Haine St-Paul, Belgium; 8 Queens University Belfast & Regional Genetics Centre, Belfast Health and Social Care Trust/City Hospital Belfast BT9 7AB Northern Ireland UK; 9 Robarts Research Institute, London, Ontario, Canada; 10 OCDEM, Radcliffe Department of Medicine, University of Oxford, Churchill Hospital, Oxford, UK; 11 Cardiology Department, Hadassah Hebrew University Medical Center, Jerusalem, Israel; 12 Department of Medicine, Center for Research, Prevention and Treatment of Atherosclerosis, Hadassah Hebrew University Medical Centre, Jerusalem, Israel; 13 North East Thames Regional Genetics Service, Great Ormond Street Hospital for Children, London, UK; 14 Consultant Lipidologist and Chemical Pathologist Director SAS Laboratory for Cardiac Biomarkers, Royal Free Hospital, London, UK; 15 Cardiovascular Trials Unit, University Department of Medicine, Central Manchester University Hospital NHS Foundation Trust, Manchester, UK; 16 Division of Laboratory Medicine, Department of Biochemistry, Royal Perth Hospital, Perth, Australia; 17 The University of Western Australia, Perth, Australia

**Keywords:** Genetics, Lipid Disorders, Diagnosis, Cardiovascular Medicine

## Abstract

**Background:**

Familial hypercholesterolaemia (FH) is an autosomal dominant disease of lipid metabolism, which leads to early coronary heart disease. Mutations in *LDLR*, *APOB* and *PCSK9* can be detected in 80% of definite FH (DFH) patients. This study aimed to identify novel FH-causing genetic variants in patients with no detectable mutation.

**Methods and results:**

Exomes of 125 unrelated DFH patients were sequenced, as part of the UK10K project. First, analysis of known FH genes identified 23 *LDLR* and two *APOB* mutations, and patients with explained causes of FH were excluded from further analysis. Second, common and rare variants in genes associated with low-density lipoprotein cholesterol (LDL-C) levels in genome-wide association study (GWAS) meta-analysis were examined. There was no clear rare variant association in LDL-C GWAS hits; however, there were 29 patients with a high LDL-C SNP score suggestive of polygenic hypercholesterolaemia. Finally, a gene-based burden test for an excess of rare (frequency <0.005) or novel variants in cases versus 1926 controls was performed, with variants with an unlikely functional effect (intronic, synonymous) filtered out.

**Conclusions:**

No major novel locus for FH was detected, with no gene having a functional variant in more than three patients; however, an excess of novel variants was found in 18 genes, of which the strongest candidates included *CH25H* and *INSIG2* (p<4.3×10^−4^ and p<3.7×10^−3^, respectively). This suggests that the genetic cause of FH in these unexplained cases is likely to be very heterogeneous, which complicates the diagnostic and novel gene discovery process.

## Introduction

Familial hypercholesterolaemia (FH (OMIM #143890)) is a genetic disorder, inherited in an autosomal dominant fashion, characterised by the defective plasma clearance of low-density lipoprotein cholesterol (LDL-C) and caused by mutations in three genes: *LDLR*, *APOB* and *PCSK9.*^1^ A recessive form of FH due to mutations in *LDLRAP1* is also known.^2^ FH is estimated to affect one in 500 individuals^3^ and if untreated leads to premature coronary heart disease (CHD).^4^ In the UK, the FH Simon Broome criteria are used for the diagnosis, which classify patients into possible FH, when adults present with total cholesterol >7.5 mmol/L or LDL-C >4.9 mmol/L, and family history of high cholesterol or premature CHD, or the more severe form—definite FH (DFH), when in addition to the above, tendon xanthomas are present in the patient or first or second degree relative.^5^ The FH mutation detection rate for DFH patients varies between 63% and 87%,^6^^–^8 suggesting that there are other genetic causes, located outside of the currently screened regions, which are yet to be identified. The importance of identifying an FH-causing variant, which has clinical utility in providing an unequivocal diagnosis,^9^ has been emphasised by the National Institute of Health and Care Excellence, which in 2008 recommended cascade testing using DNA information for finding the affected relatives of a patient.^10^ The risk of early CHD can be significantly reduced by statin treatment,^11^ and genetic information has been demonstrated to complement the management of treated patients.^12^

Of FH patients where a mutation can be found, ∼93% occur in the *LDLR* gene.^13^ The *APOB* variant (c.10580G>A, p.(Arg3527Gln)) accounts for ∼5% of UK FH cases,^7^ ^8^ ^14^ whereas a gain-of-function mutation in *PCSK9* (c.1120G>T, p.(Asp374Tyr)) can be found in roughly 1.7% of FH patients.^14^ In the past few years, several loci have been reported to cosegregate with FH in family linkage studies; however, to date, this has not led to the identification of a specific causal gene.^15^^–^17 It is likely that there are novel FH mutations located in unknown genes influencing lipid metabolism and that their discovery may contribute to the identification of novel treatment targets. In order to find novel causes of FH it was agreed that, as part of the UK10K project (http://www.uk10k.org/studies/rarediseases.html), the whole exomes of 125 unrelated DFH patients were sequenced at a high depth. We expected that an FH-causing mutation in a novel gene would be very rare accounting for fewer FH cases than the gain-of-function mutation in *PCSK9* (1.7%), since a higher frequency would have made likely its identification in previous studies. We also suspected that a proportion of patients would have polygenic hypercholesterolaemia, due to the combined impact of common LDL-C-raising SNPs.^18^

## Materials and methods

### Patients

A total of 125 unrelated patients, diagnosed as DFH using the UK Simon Broome criteria on the basis of the presence or family history of tendon xanthomas, were initially screened and shown to be negative for mutations in known FH genes (*LDLR*, *APOB*, *PCSK9* and *LDLRAP1*). All consents and local review board approvals were in accordance with the UK10K project ethical framework. The initial mutation screening methods varied and are summarised in online supplementary table S1.

### Controls

The association with FH was tested against consented 1926 UK10K samples with no lipid abnormalities (listed in online supplementary methods), sequenced in parallel, using the same sequence capture and variant calling methods (http://www.uk10k.org/studies/).

### Exome sequencing and variant calling

The whole exome sequencing was performed and processed at the Wellcome Trust Sanger Institute (Cambridge, UK) as part of the UK10K project (see online supplementary methods). CNVs were called using the ExomeDepth package for R (freely available at the Comprehensive R Archive Network).^19^

### Filtering of the variants

Variants were flagged as *rare* (frequency<0.5%) and *novel* (frequency=0) according to their frequency in publicly available databases including 1000 Genomes^20^ and National Heart, Lung, and Blood Institute (NHLBI) Exome Sequencing Project (ESP6500) (http://evs.gs.washington.edu/EVS/). In addition to the frequency filters, a *functional* flag was added, which prioritised variants that are most likely to affect a protein's function, that is, non-synonymous, stop gain, stop loss, frameshift deletions and insertions, and splice site variants.

### Burden test for association

*Rare* or *novel* variants were combined in a single gene manner and counted in cases versus 1926 controls (ie, gene by gene). A binomial test was used to assess the excess of *functional rare* and *novel* variants in cases in comparison with the controls. p Values lower than 4×10^−3^ were taken as evidence sufficient to be flagged for follow-up.

### Analysis of the variants

Variants within Tier 1 genes (*LDLR*, *APOB*, *PCSK9*, *LDLRAP1*) were assessed on the basis of their frequency, and manually by looking at their annotations in the UCL FH mutation database.^21^ Sanger sequencing was used to confirm all called mutations. Samples with known FH mutations and therefore an explained cause were removed from further analysis.

The Tier 2 list (see online supplementary table S2) consisted of genes associated with LDL-C as a lead trait in the largest (at the time) available Global Lipid Genetic Consortium (GLGC) meta-analysis of genome-wide association studies (GWASs).^22^ *Functional rare* and *novel* variants in the Tier 2 genes were compared by the burden test against non-FH controls, as one group (counts in all genes combined) and by each single gene.

### LDL-C gene score analysis

The possibility of polygenic hypercholesterolaemia in this cohort was assessed using the LDL-C gene score analysis, recently described.^18^ Most of the 12 LDL-raising GWAS SNPs are located outside of the coding regions, and thus to obtain these genotypes, methods as in the original publication were used.^18^ Gene scores were calculated by summing the weights of LDL-raising alleles provided by the GLGC (see online supplementary table S3) and the *APOE* haplotype was scored as follows: **ε**2**ε**2=−0.9, **ε**2**ε**3=−0.4, **ε**2**ε**4=−0.2, **ε**3/**ε**3=0, **ε**3**ε**4=0.1 and **ε**4**ε**4=0.2.^22^ Gene scores of a randomly selected subjects from the UK Whitehall II (WHII) study (n=3020) were used as a healthy control comparison group.^23^ Individuals with a gene score above 1.16, which was the top decile cut-off for the WHII subjects, were considered to have polygenic hypercholesterolaemia. The Welch two sample t test was used to test for an overall difference between the groups.

## Results

**Figure 1:**
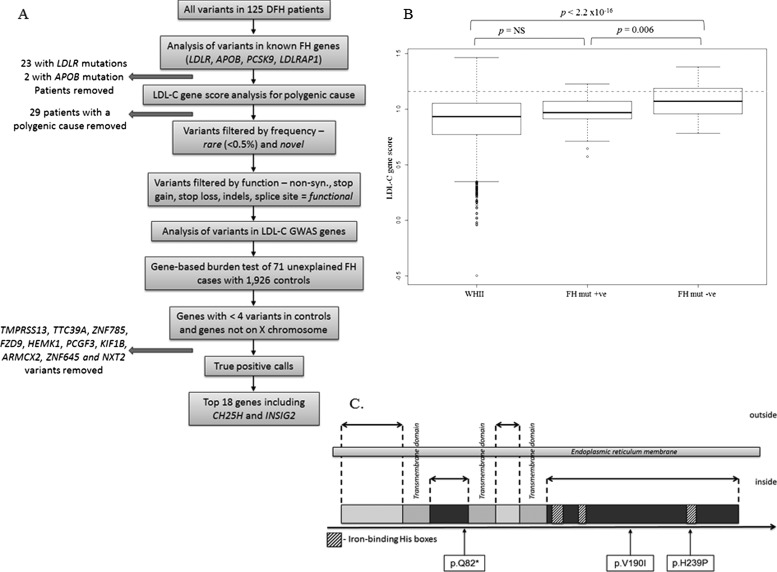
Novel familial hypercholesterolaemia (FH) gene discovery pipeline. (A) To increase the chance of detecting true FH-causing variants with a strong effect and reduce the noise, samples with a mutation in *LDLR* or *APOB* (apart from novel *APOB* variants of unknown effect) or those with a high low-density lipoprotein cholesterol (LDL-C) gene score were removed from the analysis. The remaining variants were filtered by their frequency and *functional* effect and compared against controls. Genes with more than four *novel functional* variants in controls or genes located on the X chromosome were filtered out to enhance the power of the test. The remaining variants were manually assessed and false positive calls were removed. (B) Comparison of the LDL-C SNPs score among the WHII control population (n=3020), FH mutation positive individuals (n=21) and FH mutation negative individuals (n=83) in a standard boxplot (the minimum, lower quartile, median, upper quartile and maximum). The overall difference between the groups was highly significant (ANOVA, p<2.2×10^−16^). Dashed line indicates the top decile cut-off for the WHII cohort (=1.16). A gene score was not attainable for 16 samples due to a poor DNA quality and insufficient concentration, which resulted in incomplete genotype data. (C) Schematic representation of the intronless *CH25H* gene and the localisation of novel variants identified in the FH cohort (in boxes). *CH25H* encodes an enzyme, cholesterol 25-hydroxylase, known to be spanning the endoplasmic reticulum membrane, with two domains (including the N-terminal) located outside of the membrane (in light grey), three 20 amino acid long transmembrane regions and two domains positioned inside the membrane, which contain three His boxes, essential for the catalytic activity of the enzyme.^30^

We first analysed variants in known FH genes (figure 1A) (for gene coverage see online supplementary results). For *LDLR*, 10 individuals were carrying a missense mutation, five a nonsense mutation, three had small deletions and two individuals had intronic changes known to affect splicing (see online supplementary table S4). Analysis with ExomeDepth for CNVs identified two large duplications and one deletion within the *LDLR* region (see online supplementary figure S1). For *APOB*, two individuals carried the known FH mutation, c.10580G>A (p.R3527Q), and several *novel* and cases-unique *APOB* variants, distributed across different gene exons, were identified (see online supplementary table S5). These included a recently identified mutation, p.R50W, which cosegregated with the disease.^24^ Because *APOB* is highly polymorphic, the overall number of rare variants was not significantly different in comparison with controls. *PCSK9* had the lowest mean read depth (18×), with four exons (1, 5, 9 and 10) covered less than 10× due to a high guanine and/or cytosine (GC) content (see online supplementary figure S2). There were no FH-causing variants identified in this gene. There were no homozygous or compound heterozygous calls in the *LDLRAP1* gene in any of the samples. One patient was found to be heterozygous for a previously identified frameshift mutation (c.432_433insA (p.(Ala145LysfsX26))).^25^

### LDL-C gene score analysis

Out of 109 FH samples (21 mutation positive, 88 mutation negative) with sufficient DNA for genotyping for all 12 SNPs, 31 had a gene score above the 1.16 cut-off (figure 1A), within which two samples, in addition to the high gene score, had an *LDLR* mutation, one in exon 11 (c.1690A>C (p.N564H)) found on the same allele as a 9bp deletion in exon 17 (c.2393_2401del9 (p.L799_V801del)), which has been demonstrated as not fully-penetrant.^26^ The other was a deletion of a consensus splice site at the 5′ of exon 5, c.695-6_698del, which has not been examined in vivo to confirm its likely effect on splicing.

The mean LDL-C gene score for the FH mutation negative group was 1.08, which was significantly higher than 0.90 for the WHII study (p<2.2×10^−16^), and 0.96 for the FH mutation positive group (p=0.006) (figure 1B) (for the distribution of scores see online supplementary figure S3). The overall difference between the groups was significant (analysis of variance (ANOVA), p=1.33×10^−12^). Individuals with a gene score above the top decile cut-off for the WHII subjects (>1.16), were considered to have polygenic hypercholesterolaemia and excluded from further analysis as they were unlikely to carry a single mutation of a strong effect.

### GWAS LDL-C genes

We next examined any gene identified through GWAS as being involved in determining levels of LDL-C in healthy individuals.^22^ A burden test on all *functional rare* and *novel* variants in any gene singly or in all Tier 2 genes combined showed no obvious candidate for a novel FH locus (see online supplementary table S6). In addition, there were no loss-of-function variants (ie, premature stop codon formation, loss of a stop codon, frameshift indels, CNVs) observed in these genes in any sample (n=125), or in the 71 with no identified mutation and a low gene score. There was no association of *novel functional* variants in any gene located within the several loci identified by published family linkage studies (see online supplementary table S7).

### Whole exome analysis

In all, 25 samples carrying a mutation in Tier 1 genes and 29 with the LDL-C gene score above 1.16 were removed from further analysis. To interrogate the whole exome, a burden test was performed between 71 cases and 1926 controls. There were 4407 genes with one or more *novel functional* variant in cases. In order to remove calls less likely to influence the FH phenotype and increase the power of the test, we limited further analysis only to genes where a maximum of four *novel functional* variants were seen in the controls, based on the expected prevalence of FH of 1 in 500, and therefore any gene with >4 *novel functional* variants in the controls were excluded (the original gene list is shown in online supplementary table S8). Variants in genes located on the X chromosome were removed from the final list (X chromosome genes shown in online supplementary table S8). The next step involved a visual validation of the quality of calls performed using the Human Genome 19 on the Integrative Genomic Viewer (IGV).^27^ In order to avoid false negatives, calls that were filtered out due to inadequate quality were reanalysed in genes showing excess of *novel* variants. An additional loss-of-function variant, a premature stop codon at the position c.244C>T (p.Q81*), was found in the *CH25H* gene in an FH patient sample with a low LDL-C SNP score. After adjusting for the false positives and false negatives, *CH25H* remained the top gene (p<4.3×10^−4^) with three variants in the cases and two in the controls (table 1). To examine the prevalence of nonsense variants in *CH25H* in public data sources, we analysed the NHLBI ESP database and found one nonsense allele (c.638delT) in 6503 individuals (Minor Allele Frequency (MAF)=0.00008), which was significantly lower than in the FH group (MAF=0.0003, p<1.5×10^−3^).

**Table 1:** Summary of genes and their variants which show an excess of *novel functional* variants in FH cases (n=71) in comparison with controls (n=1926)

Gene		Ch	Number of variants in cases (n=71)	Number of variants in controls (n=1926)		p Value
***CH25H***		**10**	**3**	**2**		**4.3×10^−4^**
	Cases		ENST00000371852:exon1:c.G568A:p.V190I; exon1:c.A716C:p.H239P; exon1:c.C244T:p.Q82X			
	Controls		ENST00000371852:exon1:c.T742G:p.C248G; exon1:c.C590A:p.P197Q			
***HSPB7***		**1**	**2**	**0**		**1.3×10^−3^**
	Cases		2X ENST00000311890:exon2:c.199+7G>A			
	Controls		None			
***KLRC1***		**12**	**2**	**0**		**1.3×10^−3^**
	Cases		ENST00000544822:exon5:c.G333C:p.Q111H; exon3:c.C178T:p.H60Y			
	Controls		None			
***MOAP1***		**14**	**3**	**4**		**1.4×10^−3^**
	Cases		ENST00000556883:exon2:c.C707T:p.A236V; exon2:c.G476C:p.C159S; exon2:c.A182G:p.N61S			
	Controls		ENST00000556883:exon2:c.C655G:p.R219G; exon2:c.C627A:p.S209R; exon2:c.C264G:p.I88M; exon2:c.A919G:p.I307V			
***RBM25***		**14**	**3**	**4**		**1.4×10^−3^**
	Cases		ENST00000261973:exon6:c.A454T:p.I152F; exon2:c.T50C:p.L17P; exon11:c.C1364A:p.A455D			
	Controls		ENST00000261973:exon7:c.C671T:p.A224V; exon11:c.A1273G:p.R425G; exon18:c.G2392A:p.V798I; exon2:c.T7C:p.F3L			
***ANP32E***		**1**	**2**	**1**		**3.7×10^−3^**
	Cases		ENST00000436748:exon3:c.G227C:p.S76T; ENST00000533654:exon4:c.A434G:p.K145R			
	Controls		ENST00000436748:exon6:c.G629T:p.R210L			
***CABP5***		**19**	**2**	**1**		**3.7×10^−3^**
	Cases		ENST00000293255:exon4:c.C281A:p.T94N; exon3:c.G201A:p.M67I			
	Controls		ENST00000293255:exon3:c.A169C:p.M57L			
***CELA2B***		**1**	**2**	**1**		**3.7×10^−3^**
	Cases		ENST00000375910:exon6:c.G576A:p.W192X; ENST00000422901:exon3:c.G271A:p.G91R			
	Controls		ENST00000375910:exon7:c.T739C:p.Y247H			
***INSIG2***		**2**	**2**	**1**		**3.7×10^−3^**
	Cases		ENST00000245787:exon2:c.T89C:p.I30T; exon2:c.C236T:p.T79M			
	Controls		ENST00000245787:exon4:c.G376A:p.D126N			
***KCTD7***		**7**	**2**	**1**		**3.7×10^−3^**
	Cases		ENST00000275532:exon4:c.G814A:p.V272M; exon4:c.C758T:p.S253L			
	Controls		ENST00000275532:exon4:c.G506A:p.R169Q			
***MRO***		**18**	**2**	**1**		**3.7×10^−3^**
	Cases		ENST00000436348:exon5:c.G578A:p.R193Q; exon5:c.G565A:p.V189I			
	Controls		ENST00000436348:exon3:c.A223G:p.S75G			
***NR2E1***		**6**	**2**	**1**		**3.7×10^−3^**
	Cases		ENST00000368983:exon1:c.G136A:p.G46S; exon5:c.A634G:p.M212V			
	Controls		ENST00000368983:exon7:c.G1000A:p.V334I			
***PABPC1***		**8**	**2**	**1**		**3.7×10^−3^**
	Cases		ENST00000318607:exon9:c.A1250C:p.Q417P;exon10:c.G1364A:p.R455H			
	Controls		ENST00000523555:exon3:c.226+3A>G			
***PODXL***		**7**	**2**	**1**		**3.7×10^−3^**
	Cases		ENST00000537928:exon3:c.G821A:p.R274K; exon5:c.A992G:p.H331R			
	Controls		ENST00000537928:exon8:c.C1246G:p.Q416E			
***PUS3***		**11**	**2**	**1**		**3.7×10^−3^**
	Cases		ENST00000530811:exon1:c.T74C:p.V25A; exon2:c.T824C:p.L275P			
	Controls		ENST00000530811:exon4:c.945-8T>C			
***TXNDC15***		**5**	**2**	**1**		**3.7×10^−3^**
	Cases		ENST00000511070:exon2:c.C130T:p.R44W; ENST00000507024:exon2:c.G91A:p.A31T			
	Controls		ENST00000358387:exon2:c.G534C:p.E178D			
***WDR89***		**14**	**2**	**1**		**3.7×10^−3^**
	Cases		ENST00000394942:exon2:c.T821C:p.L274S; exon2:c.A553G:p.M185V			
	Controls		ENST00000394942:exon2:c.A860G:p.D287G			
***ZNF720***		**16**	**2**	**1**		**3.7×10^−3^**
	Cases		ENST00000398696:exon2:c.T508G:p.L170V; exon2:c.A29G:p.H10R			
	Controls		ENST00000399681:exon6:c.A893G:p.H298R			

Ch, chromosome; FH, familial hypercholesterolaemia.

### *CH25H* and *INSIG2* variants

*CH25H* codes for cholesterol 25-hydroxylase, known to catalyse the formation of the oxysterol—25-hydroxycholesterol (25-HC) (9). The *INSIG2* gene, which also exhibited an excess of *novel functional* variants in the FH cohort in comparison with the controls (p=3.7×10^−3^) (table 1), has been demonstrated to regulate the activity of Sterol Regulatory Element-Binding Protein (SREBPs), a family of major lipid metabolism transcription factors, via direct biding of 25-HC.^28^ Thus, both genes, *CH25H* and *INSIG2*, are involved in the same pathway of cholesterol metabolism. There were three heterozygous variants found in *CH25H*, all confirmed by Sanger sequencing (see online supplementary figure S4), of which one leads to a formation of a premature stop codon at residue 81, predicted to have a damaging effect on the protein; the second affects a well-conserved residue across species, c.568G>A (p.V190I); and the third, c.716A>C (p.H239P), alters one of the crucial residues of the His Box 3 domain, known to play a crucial role, together with His Boxes 1 and 2, in the catalytic activity of *CH25H*^29^ (figure 1C). Two *novel functional* variants were found in the control cohort, both being non-synonymous (p.P197Q and p.C248G). The p.P197Q is located in a conserved region of the protein; however, it is predicted as tolerated/benign/neutral by SIFT/PolyPhen/Mutation Taster. The p.C248G variant affects a residue that is not conserved.^30^

Sanger sequencing also confirmed two *novel functional* variants in the *INSIG2* gene called in the cases, both non-synonymous changes (see online supplementary figure S5). A mutation prediction report generated by Project HOPE^31^ highlighted that the c.89T>C (p.I30T) variant will cause an empty space in the core of INSIG2 because of the size differences between the wild type Isoleucine and the smaller mutant—Threonine. The other variant, c.236C>T (p.T79M), located in the transmembrane domain of INSIG2, is predicted to have an effect on the hydrophobic interactions within the core of the protein or with the membrane lipids, because the mutant Methionine is more hydrophobic than the wild type Threonine. One rare missense variant was found in *INSIG2* in the controls (p.D126N), which was predicted as tolerated/probably damaging/disease causing (by SIFT/PolyPhen/Mutation Taster).

## Discussion

In this study, we have identified 25 mutations in known FH genes (23 in *LDLR* and two in *APOB*), which were missed by the current screening protocol. Because the sequencing coverage of the *PCSK9* gene was lower than for *LDLR* and *APOB*, we cannot rule out that there may have been undetected mutations in this gene also. This finding confirmed that *LDLR* locus is highly heterogeneous and mutations within this gene account for the majority of FH causes. The issue of genetic misdiagnosis and the need for an update of current screening methods have been previously discussed.^32^ In addition to the known FH mutations, we identified six novel *APOB* variants, distributed across different exons, in five patients, which included the recently examined p.R50W variant.^33^ The pathogenicity of these variants remains to be tested. Most of the current mutation screening strategies for FH are focused on a selected region of exon 26 of *APOB*, because of its established function;^34^ however, the whole exome sequencing enabled us to analyse the entire coding sequence of the gene, by which we found novel variants unique to the FH cohort.

### Polygenic hypercholesterolaemia

The cumulative effect of common LDL-raising alleles in genes identified by GWAS was shown to be the likely cause of high LDL-C in a significant proportion (27%) of the examined patients. A gene score above the top decile for a healthy population cut-off (1.16) was also observed in two patients with considerably mild *LDLR* mutations, which demonstrates that common polymorphisms can contribute to the presentation of an individual carrying a mild effect FH mutation with LDL-C levels above the diagnostic threshold.

### GWAS LDL-C genes

Since common variants in the LDL-C-associated GWAS genes were found to be important in the FH pathogenesis, we looked for evidence that rare variants in these genes were causing FH. *Rare* and *novel functional* variants in genes associated with LDL-C levels in the GWAS meta-analysis were not significantly over-represented in the FH cohort, when compared with controls. This suggests that rare variants that have a major effect on function in these genes known to have common LDL-C variants of modest effect are unlikely to be a common cause of FH.

### *CH25H* and *INSIG2* variants

Genes *CH25H* and *INSIG2* are the strongest candidates for novel FH loci among the final 18 genes, showing an excess of *novel functional* variants, based on the available reports on functions of the proteins for which they code. *CH25H* encodes 25-cholesterol hydroxylase, which catalyses the formation of 25-HC from cholesterol. The gene is located in close proximity to the *LIPA* gene in which mutations were recently found in patients with autosomal recessive FH phenotype.^35^ It has been demonstrated that both cholesterol and 25-HC can regulate the function of SREBP, a transcription factor known to regulate the expression of several key players in the lipid metabolism.^36^ ^37^ It is known that the regulation of SREBP activity depends on binding of 25-HC to INSIG2, encoded by the *INSIG2* gene.^28^ The recently updated GLGC GWAS study with >180 000 individuals has identified an association at the genome-wide level of LDL-C with an *INSIG2* gene variant (rs10490626, MAF=0.08).^38^

The *CH25H* variants identified in this study have not been observed in 1000 Genomes, 6500ESP and 69CG or the 1926 control exomes. We therefore decided to sequence the gene in an additional cohort of 150 mutation negative FH patients with a low gene score, but no additional amino acid changes were identified.

A detailed literature search and gene ontology analysis of the remaining 16 most significant genes did not reveal any clear association with lipid metabolism. We suspect that the majority of these associations are false positives, and that increasing the number of DFH cases would help to reduce the number of chance signals. It is also possible that some of the top genes are indeed affecting the plasma clearance of LDL-C; however, their biology is yet to be understood.

There are a number of limitations to our study. An alternative study design would be to use Next Generation Sequencing (NGS) of relatives (or trios) from selected families with clear autosomal dominant hypercholesterolaemia. The UK10K study only allowed for 125 subjects with FH to be included, and we calculated that, if we selected 125 singleton no-mutation patients with a clinical diagnosis of DFH, we would expect four to carry a shared mutated locus leading to the defective plasma clearance of LDL cholesterol. The power of the study is clearly dependent on the number of singletons included, with the idea that any identified candidate locus would be sequenced in the family members of the affected proband. While a group of singletons may be genetically heterogeneous, the use of the ‘burden’ analysis and not a single-variant test means that heterogeneity should not reduce power to detect a novel FH-causing gene. Another limitation is that we did not have lipid profile information for individuals in the control comparison cohort, only their rare disease phenotype status, which did not overlap with FH pathogenesis. The possibility that the control cohort includes FH-affected individuals was considered. Assuming that the prevalence of FH is 1/500, we would expect by chance to find ∼4 individuals in this cohort carrying an FH-causing mutation. We have analysed variants in *LDLR*, *APOB* and *PCSK9* in the control cohort and identified three *LDLR* and two *APOB* mutations as incidental findings, which was similar to the expected FH frequency of one in 500. We have also allowed for this prevalence in the control comparison cohort by using a frequency cut-off of four novel gene functional variants in controls, in case any of the novel variants identified in FH cases were also present in the controls. A final limitation is that it is possible that some of the identified variants in the 18 genes in table 1 may be technical false positives, since only for the *CH25H* and *INSIG2* genes were all variants confirmed by Sanger Sequencing, However, to be as certain as possible using bioinformatics that the variants we observed are not false positives, for all these variants we included a visual validation of the quality of calls performed using the Human Genome 19 on the IGV.^27^

In summary, in 125 DFH unrelated patients without an identified mutation by conventional screening methods, analyses identified 25 disease-causing variants in already known FH loci, as well as six previously unreported *APOB* variants in five patients. LDL-C gene score analysis found that 31 (29 mutation negative) patients had an SNP score in the top decile of the general population and therefore had a definite polygenic aetiology, and an additional five had a potential functional variant in *CH25H* or *INSIG2*. This means that the explanation for the FH phenotype is still lacking in 50% of the patients, suggesting that some causal variants may have been missed at different stages of the data processing or analysis. The variant calling pipeline used for this study was carefully optimised for the majority of the exome regions, though some calls in poorly covered regions could be missed. There is a possibility that there are genetic causes located outside of the protein coding region, affecting protein expression, posttranscriptional stability or altering gene splicing. Also, it is possible that the LDL-C gene score cut-off of 1.16 for polygenic hypercholesterolaemia is too stringent. Thus, using the 9th decile cut-off of 1.08, in which a 41% of WHII individuals had LDL-C above the 4.9 mmol/L (mean LDL-C=4.68±1.05 mmol/L) FH diagnostic level, could be more appropriate. By doing so, the phenotype would be explained in an additional nine mutation-negative patients. A polygenic explanation in additional subjects might also be achieved if SNPs in recently identified LDL-C-raising loci^38^ were included in the score. Finally, because the burden test results are dependent on the number of associated variants and variants diluting the signal, it is possible that novel FH mutations are located in a highly polymorphic gene, in which it is difficult to pick up the true mutation.

Thus, overall, no major novel locus for FH was detected, with no gene having a functional variant in more than three patients. This suggests that the genetic cause of FH in these unexplained cases is likely to be very heterogeneous, which complicates the novel gene discovery and diagnostic process.

## Supplementary Material

Web supplement
