# Supplementary material for: Whole exome sequencing of familial hypercholesterolaemia patients negative for LDLR/APOB/PCSK9 mutations
Source: J Med Genet. 2014 Aug;51(8):537–44. doi: 10.1136/jmedgenet-2014-102405 (PMC4112429; doi:10.1136/jmedgenet-2014-102405)
Supplement: Web supplement [file jmedgenet-2014-102405-s1.pdf]

SUPPLEMENTARY METHODS

Control samples cohorts:

UK10K_NEURO_ABERDEEN (n=387)
UK10K_NEURO_ASD_GALLAGHER (n=75)
UK10K_NEURO_EDINBURGH (n=233)
UK10K_NEURO_GURLING (n=48)
UK10K_NEURO_IOP_COLLIER (n=172)
UK10K_NEURO_MUIR (n=166)
UK10K_OBESITY_GS (n=421)
UK10K_OBESITY_TWINSUK (n=67)
UK10K_RARE_CILIOPATHIES (n=121)
UK10K_RARE_NEUROMUSCULAR (n=114)
UK10K_RARE_THYROID (n=122)

For more details see http://www.uk10k.org/studies/ .

Whole exome sequencing

Genomic DNA (1–3 μg), extracted from blood (*1*), was sheared to 100–400 bp using a Covaris E210 or LE220 (Covaris, Woburn, Massachusetts, USA). Sheared DNA was subjected to Illumina paired-end DNA library preparation and enriched for target sequences (Agilent Technologies, Santa Clara, CA, USA; Human All Exon 50 Mb - ELID S02972011) according to the manufacturer's recommendations (Agilent Technologies, Santa Clara, CA, USA; SureSelectXT Automated Target Enrichment for Illumina Paired-End Multiplexed Sequencing). Enriched libraries were sequenced (eight samples over two lines) using the HiSeq 2000 platform (Illumina) as paired-end 75 base reads according to the manufacturer's protocol.

Variant calling

Calls were made using samtools/bcftools version 0.1.19-3-g4b70907 from all UK10K per-sample exome BAMs split by chromosome. A BCF file was created with samtools mpileup, calculating genotype likelihoods for every site in the bait (+/-100bp) regions file then variants (SNPs and Indels) were called by bcftools.

SUPPLEMENTARY RESULTS

Gene coverage

The overall mean coverage of *LDLR*, *APOB*, *PCSK9* and *LDLRAP1* ranged from 42x (*LDLR*), to 18x (*PCSK9*), with the first and the last exons of a gene having the lowest coverage. The read depth was highly dependent on the GC content of an exon (regression p $=4.9 \times 10^{-14}$) (Figure S2). Exons of the *APOB* had the highest average read depth among Tier 1 genes (58x).

LDL-C SNPs score

The distribution of LDL-C SNPs scores in FH mutation negative patients and in the healthy WHII population was as shown in Figure S3.

The *APOE* ε2ε2 genotype was not observed among the genotyped patients. There were two individuals with the ε2ε3 genotype, both having an *LDLR* mutation. Five patients had the ε4ε4 isoform.

Figure S1.
Copy Number Variants (CNVs) in *LDLR* gene. A: Heterozygous duplication of exons 3 to 8. B: Heterozygous deletion of exons 11 and 12. C: Heterozygous duplication of exons 13 to 15. All identified by ExomeDepth in the exome sequencing data. The crosses show the ratio of observed/expected number of reads for the test sample. The grey shaded region shows the estimated 99% confidence interval for this observed ratio in the absence of CNV call. The presence of contiguous exons with read count ratio located outside of the confidence interval is indicative of a heterozygous deletion or duplication in a sample. Exons 1 and 18 were excluded from the analysis (not shown on the graph) as they did not reach the threshold of 100 for the total number of reads. All CNVs were confirmed by MLPA experiment. The deletion of exons 11-12 and duplication of exons 13-15 both lead to a frame shift. The duplication of exons 3-8 leads to elongated peptide and it has been previously found in FH patients (*2*).

A B C

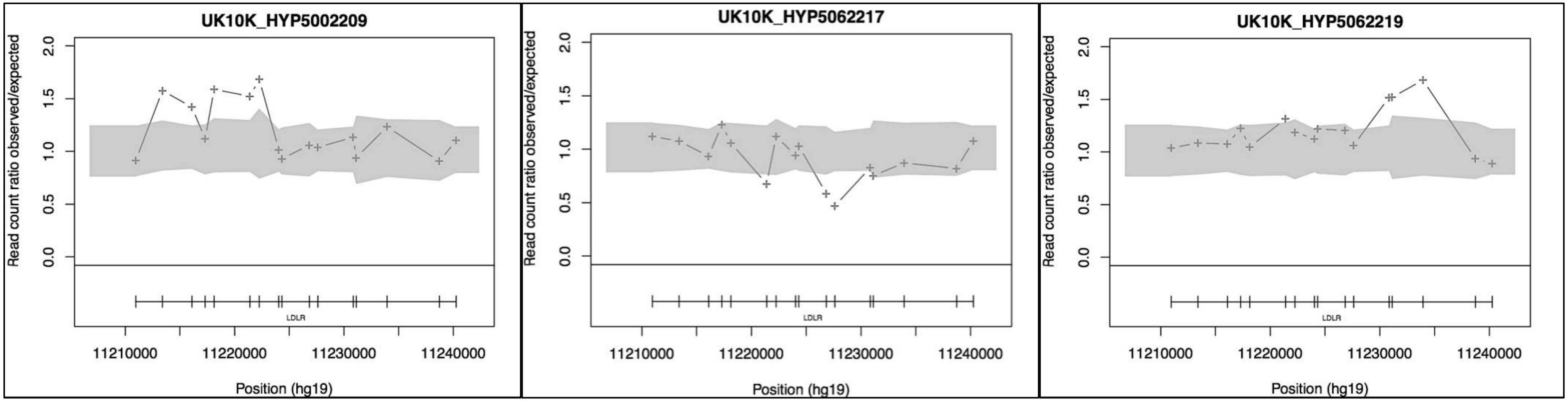

Figure S2.
The negative correlation of the median read depth and the GC content for each targeted exon of the four FH genes (*LDLR*, *APOB*, *PCSK9* and *LDLRAP1*).

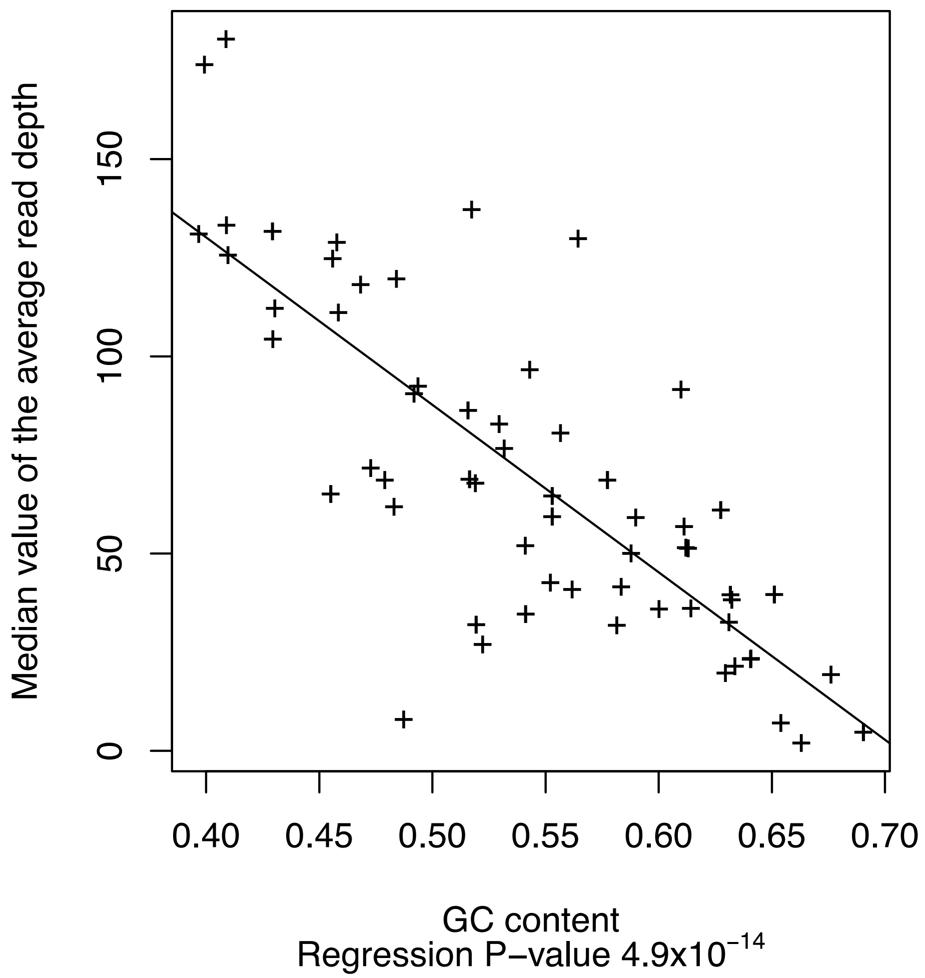

Figure S3.
Distribution of the LDL-C SNPs score in mutation negative DFH patients (in purple) and in the healthy WHII cohort (in grey). Red line indicates the LDL-C score top decile cutoff for WHII (=1.16).

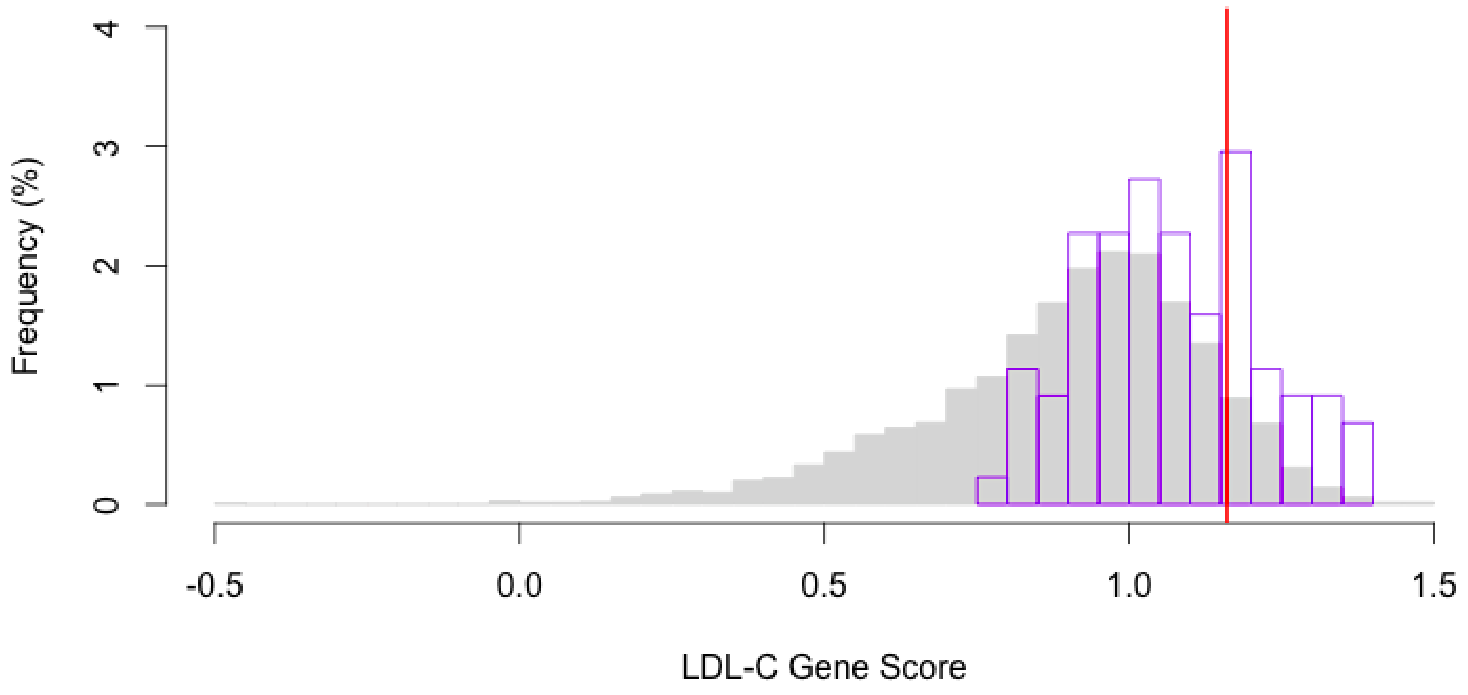

Figure S4.
Sanger sequencing confirmation of novel *CH25H* variants. Primers used for the amplification of the region are highlighted in blue and in purple

CH25H sequencing (order #410822401)

```
90966631 TYGTGTGGCACCTGCTGCACCACAAGGTGCCCTGGYTGTACCGCACCTTYCACAAGGTGC 90966572
90966571 ACCACCRGAACTCGTCCTCGTTCGCRCTGGCAACGCAGTATATGAGCGTCTGGGAACTGT 90966512
90966511 TTTCTTTGGGYTTCTTCGACATGATGAACGTCACACTGCTYGGGTGCCACCCGCTCACCA 90966452
90966451 CCCTGACCTTCCACGTGGTCAACATCTGKCTTTCCGTGGAGGWCCACTCCGGCTACAAYT 90966392
90966391 TCCCTTGGTCCACTCACAGACTGGTGCCCTTCGGGTGGTACGGGGGTGTGGTRCACCACG 90966332
90966331 ACCTGCATCACTCTCACTTTAACTGCAACTTCGCTCCRTACTTTACACACTGGGACAAAA 90966272
90966271 YACTGGGAACRCTGCGRACTGCATCTGTCCCAGCGCRRTGATGTGGCTGCGGTGGGTGCC 90966212
90966211 CCTAAGAMTCGGGACTGCTRTGCCTTTCACACTTGAATGAAGAGAAACACCTGAGCTATA 90966152
90966151 TATTTTTTTAAAGCAACTAACTTATTRCTTTATGTTTATCTATGAAAACCATAGATAAAA 90966092
90966091 TCTGATGCATTTTTGTAATCTGACAAAGTAATTTACATACTGTTTGTGTATCAATACAAT 90966032
```

*Primers:
CH25H_01F / CH25H_02R

p.H239P (c.716A>C) (UK10K_HYP5231652)

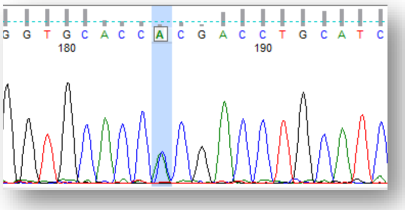

p.V190I (c.568G>A) (UK10K_HYP5231677)

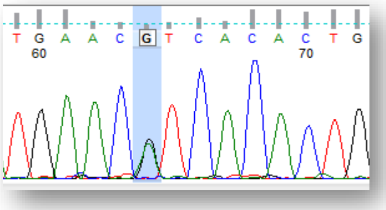

WT (UK10K_HYP5231677)

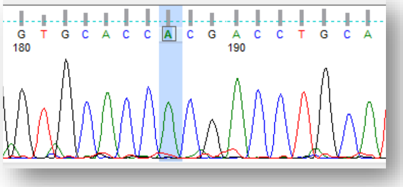

WT (UK10K_HYP5231652)

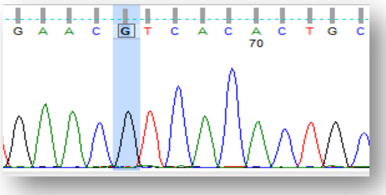

CH25H sequencing (order #4108796)

```
90966991 TGCAGCCCCTCTGGGACCACCTGAGGAGCTGGGAGGCCCTCCTACAGTCGCCCTTCTTCC 90966932
90966931 CGGTCATCTTCTCCATCACCACATACRTRGGCTTTTGCCTGCCCTTCGTGGTCCTGGATA 90966872
90966871 TCCTGTGCTCCTGGGTGCCCGCCCTGCGGCGCTACAAGATCCAYCCTGACTTCTCGCCAT 90966812
90966811 CCGCGCAGCAGCTGCTACCTTGCCTGVGRCAGACCCTCTACCAGCAKGTGATGTTTGTGT 90966752
90966751 TCCCCGTRACGCTGMTGCAYTGGGCCYGCAGCCCGGCCCTCCTGCCCCACGAAGCTCCCG 90966692
90966691 AGCTGCTCCTGCTGCTGCACCACATCCTGTTCTGCCTGCYACTCTTCGACATGGAGTTCT 90966632
90966631 TYGTGTGGCACCTGCTGCACCACAAGGTGCCCTGGYTGTACCGCACCTTYCACAAGGTGC 90966572
90966571 ACCACCRGAACTCGTCCTCGTTCGCRCTGGCAACGCAGTATATGAGCGTCTGGGAACTGT 90966512
90966511 TTTCTTTGGGYTTCTTCGACATGATGAACGTCACACTGCTYGGGTGCCACCCGCTCACCA 90966452
90966451 CCCTGACCTTCCACGTGGTCAACATCTGKCTTTCCGTGGAGGWCCACTCCGGCTACAAYT 90966392
90966391 TCCCTTGGTCCACTCACAGACTGGTGCCCTTCGGGTGGTACGGGGGTGTGGTRCACCACG 90966332
90966331 ACCTGCATCACTCTCACTTTAACTGCAACTTCGCTCCRTACTTTACACACTGGGACAAAA 90966272
90966271 YACTGGGAACRCTGCGRACTGCATCTGTCCCAGCGCRRTGATGTGGCTGCGGTGGGTGCC 90966212
```

*Primers:
CH25H _03F / CH25H_04R

p.A80A (c.243G>T) and p.Q81* (c.244C>T) (UK10K_HYP5159267)

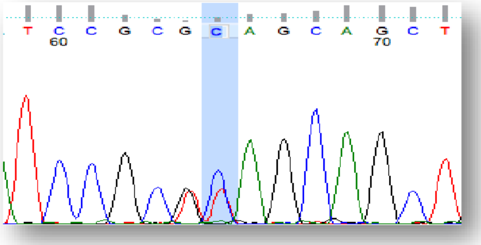

WT (UK10K_HYP5002218)

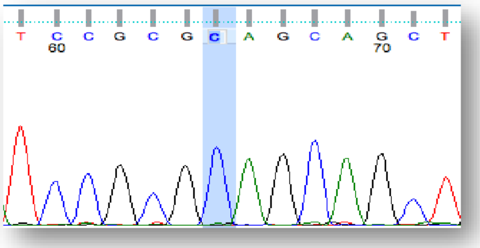

Figure S5.
Sanger sequencing confirmation of novel INSIG2 variants. Primers used for the amplification of the region are highlighted in yellow.

INSIG2 sequencing (order #4103758)

```
118853948 ATCATGTATTAGATACACATTAATTTCTTTTTTCTTATCTCTTGCAGGATTTCTGGTAGG 118854007
118854008 TCCTACTTTAGGACAAGATGTGGTACCGTTGAAGCGTCAGTCTTTGATTCACAGACAGTT 118854067
118854068 GAGCTTTTCAGCTGGGAAGCCTTTCCATTTTTTTTTTTTTTAACGGCTTTCTGAACCTATG 118854127
118854128 AAACCATGGCAGAAGGAGAGACAGAGTCACCTGGGCCCAAAAAGTGTGGCCCATATATTT 118854187
118854188 CATCTGTCACTAGCCAGAGTGTGAACTTGATGATTCGAGGAGTAGTGCTATTTTTTATTG 118854247
118854248 GAGTATTTCTTGCATTAGTGTTAAATTTACTTCAGATTCAGAGAAATGTGACGCTCTTTC 118854307
118854308 CACCTGATGTGATTGCAAGCATCTTTTCTTCTGCATGGTGGGTACCCCCATGCTGTGGCA 118854367
118854368 CGGCTTCAGGTATGTGTAGGATGTTTCTGTAATGCTTAGAAAGGAAATAGGGTAAATGAG 118854427
118854428 TATGGACGTTGTCTGAGCAATAAACCTTTTTAAAAAAGAAAATATATTTATTGAGATATA 118854487
118854488 ATTTAGGTATAATACACTGGACCCGTTTGAATTGAACAACTTGATGTGTTTAGGCAAATG 118854547
```

WT (UK10K_HYP5002210)

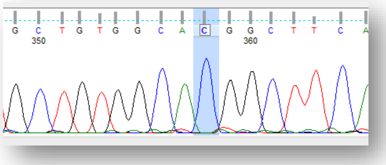

p.I30T (c.89T>C) (UK10K_HYP5002210)

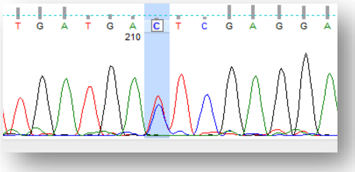

p.T79M (c.236C>T) (UK10K_HYP5231650)

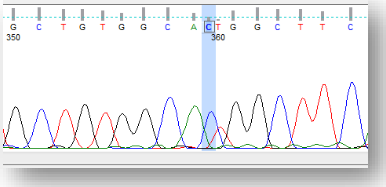

WT (UK10K_HYP5231650)

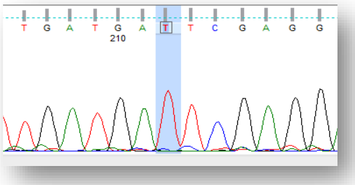

*Primers:
INSIG2_01F / INSIG2_02R

Table S1.
Summary of methods used for the initial FH mutation screening.

BATCH	UK10K ID	Original study cohort	Ref	*LDLR*	MLPA of *LDLR*	*APOB*	*PCSK9*
4	UK10K_HYP5231659	Australian FH	(*3*)	all exons and promoter by Big Dye Terminator chemistry (Applied Biosystems) sequencing	yes	fragment of exon 26, and exon 29 by Big Dye Terminator chemistry (Applied Biosystems) sequencing	exon 7 by Big Dye Terminator chemistry (Applied Biosystems) sequencing
4	UK10K_HYP5231660	Australian FH					
4	UK10K_HYP5231661	Australian FH					
4	UK10K_HYP5231662	Australian FH					
4	UK10K_HYP5231663	Australian FH					
4	UK10K_HYP5231664	Australian FH					
4	UK10K_HYP5231665	Australian FH					
4	UK10K_HYP5231666	Australian FH					
4	UK10K_HYP5231667	Australian FH					
4	UK10K_HYP5231668	Australian FH					
4	UK10K_HYP5231669	Australian FH					
4	UK10K_HYP5231670	Australian FH					
4	UK10K_HYP5231671	Australian FH					
4	UK10K_HYP5231672	Australian FH					
4	UK10K_HYP5231673	Australian FH					
4	UK10K_HYP5231674	Australian FH					
4	UK10K_HYP5231675	Australian FH					
5	UK10K_HYP5269604	Israeli FH	-	all exons and promoterby SSCP	no	APOB fragment of exon 26 by SSCP	no
5	UK10K_HYP5269605	Israeli FH					
5	UK10K_HYP5269606	Israeli FH					
5	UK10K_HYP5269607	Israeli FH					
5	UK10K_HYP5269608	Israeli FH					
5	UK10K_HYP5269609	Israeli FH					
7	UK10K_HYP5358903	Israeli FH					
7	UK10K_HYP5358904	Israeli FH					
7	UK10K_HYP5358905	Israeli FH					
7	UK10K_HYP5358906	Israeli FH					
4	UK10K_HYP5231679	Italy FH	(*4*)	all exons and	yes	c.9216 to	all exons by

4	UK10K_HYP5231676	Italy FH		promoter by Sanger sequencing		c.11788 +152 nt of intron 26 by Sanger sequencing	Sanger sequencing
4	UK10K_HYP5231677	Italy FH					
4	UK10K_HYP5231678	Italy FH					
5	UK10K_HYP5269570	Northern Ireland FH	(*5*)	all exons and promoter by TTGE/DDGE	yes	RFLP for p.R3527Q	Exon 7 by Sanger sequencing
5	UK10K_HYP5269571	Northern Ireland FH					
5	UK10K_HYP5269572	Northern Ireland FH					
5	UK10K_HYP5269573	Northern Ireland FH					
5	UK10K_HYP5269574	Northern Ireland FH					
5	UK10K_HYP5269575	Northern Ireland FH					
5	UK10K_HYP5269576	Northern Ireland FH					
5	UK10K_HYP5269577	Northern Ireland FH					
5	UK10K_HYP5269578	Northern Ireland FH					
5	UK10K_HYP5269581	Northern Ireland FH					
3	UK10K_HYP5159271	Oxford FH	(*6*)	all exons and promoter by HRM	yes	fragment of exon 26 by HRM and ARMS	ARMS for p.D374Y
3	UK10K_HYP5159272	Oxford FH					
3	UK10K_HYP5159273	Oxford FH					
3	UK10K_HYP5159274	Oxford FH					
3	UK10K_HYP5159275	Oxford FH					
4	UK10K_HYP5231650	Oxford FH					
4	UK10K_HYP5231651	Oxford FH					
4	UK10K_HYP5231652	Oxford FH					
4	UK10K_HYP5231653	Oxford FH					
4	UK10K_HYP5231654	Oxford FH					
4	UK10K_HYP5231655	Oxford FH					
4	UK10K_HYP5231656	Oxford FH					
4	UK10K_HYP5231657	Oxford FH					
4	UK10K_HYP5231658	Oxford FH					
3	UK10K_HYP5159266	RFH	(*7*)	all exons and promoter by SSCP or Sanger sequencing	yes	ARMS for p.R3527Q	ARMS for p.D374Y
3	UK10K_HYP5159267	RFH					
3	UK10K_HYP5159268	RFH					
3	UK10K_HYP5159269	RFH					
3	UK10K_HYP5159270	RFH					
1	UK10K_HYP5002209	SBBHF	(*8-*	all exons and	yes	RFLP for	all exons by HRM

1	UK10K_HYP5002210	SBBHF	*11*)	promoter by HRM		p.R3527Q		
1	UK10K_HYP5002211	SBBHF						
1	UK10K_HYP5002212	SBBHF						
1	UK10K_HYP5002213	SBBHF						
1	UK10K_HYP5002214	SBBHF						
1	UK10K_HYP5002215	SBBHF						
1	UK10K_HYP5002216	SBBHF						
1	UK10K_HYP5002217	SBBHF						
1	UK10K_HYP5002218	SBBHF						
1	UK10K_HYP5002219	SBBHF						
1	UK10K_HYP5002220	SBBHF						
1	UK10K_HYP5002221	SBBHF						
1	UK10K_HYP5002222	SBBHF						
1	UK10K_HYP5002223	SBBHF						
1	UK10K_HYP5002224	SBBHF						
1	UK10K_HYP5002225	SBBHF						
1	UK10K_HYP5002226	SBBHF						
1	UK10K_HYP5002227	SBBHF						
1	UK10K_HYP5002228	SBBHF						
1	UK10K_HYP5002229	SBBHF						
1	UK10K_HYP5002230	SBBHF						
1	UK10K_HYP5002231	SBBHF						
1	UK10K_HYP5002232	SBBHF						
2	UK10K_HYP5062209	SBBHF						
2	UK10K_HYP5062210	SBBHF						
2	UK10K_HYP5062211	SBBHF						
2	UK10K_HYP5062212	SBBHF						
2	UK10K_HYP5062213	SBBHF						
2	UK10K_HYP5062214	SBBHF						
2	UK10K_HYP5062215	SBBHF						
2	UK10K_HYP5062216	SBBHF						
2	UK10K_HYP5062217	SBBHF						
2	UK10K_HYP5062218	SBBHF						

2	UK10K_HYP5062219	SBBHF					
2	UK10K_HYP5062220	SBBHF					
2	UK10K_HYP5062221	SBBHF					
2	UK10K_HYP5062222	SBBHF					
2	UK10K_HYP5062223	SBBHF					
2	UK10K_HYP5062224	SBBHF					
2	UK10K_HYP5062225	SBBHF					
2	UK10K_HYP5062226	SBBHF					
2	UK10K_HYP5062227	SBBHF					
2	UK10K_HYP5062228	SBBHF					
2	UK10K_HYP5062229	SBBHF					
2	UK10K_HYP5062230	SBBHF					
2	UK10K_HYP5062231	SBBHF					
2	UK10K_HYP5062232	SBBHF					
5	UK10K_HYP5269585	SBBHF					
5	UK10K_HYP5269589	SBBHF					
5	UK10K_HYP5269595	SBBHF					
5	UK10K_HYP5269597	SBBHF					
5	UK10K_HYP5269598	SBBHF					
5	UK10K_HYP5269601	SBBHF					
5	UK10K_HYP5269602	SBBHF					
6	UK10K_HYP5315266	SBBHF					
6	UK10K_HYP5315268	SBBHF					
6	UK10K_HYP5315271	SBBHF					
6	UK10K_HYP5315273	SBBHF					
6	UK10K_HYP5315275	SBBHF					
7	UK10K_HYP5358898	SBBHF					
7	UK10K_HYP5358899	SBBHF					
7	UK10K_HYP5358900	SBBHF					
7	UK10K_HYP5358901	SBBHF					
7	UK10K_HYP5358902	SBBHF					

Table S2.

Tier 2 candidate genes – LDL-C (lead trait) associated loci from Teslovich et al. GWAS meta-analysis (either a plausible biological candidate gene in the locus or the nearest annotated gene to the lead SNP) (*12*). Where associated SNP was located in a gene cluster, other genes in the region were included.

**Gene ID**
ABCG5
ABCG8
ABO
APOE
APOC1(APOE locus)
TOMM40 (APOE locus)
PVRL2 (APOE locus)
HFE
LPA
MYLIP
NYNRIN
OSBPL7
SORT1
CELSR2 (SORT1 locus)
PSRC1 (SORT1 locus)
ST3GAL4
DCPS (ST3GAL4 locus)
KIRREL3
TOP1
PLCG1 (TOP1 locus)
ZHX3 (TOP1 locus)
LPIN3(TOP1 locus)
PLEC1
PARP10 (PLEC locus)
GRINA (PLEC locus)
SPATC1 (PLEC locus)
OPLAH (PLEC locus)
EXOSC4 (PLEC locus)
GPAA1 (PLEC locus)
KIAA1875 (PLEC locus)
CYC1 (PLEC locus)
SHARPIN (PLEC locus)
MAF1 (PLEC locus)

Table S3.
The top LDL-rising SNPs and their effects (as reported by the GLGC) used for the LDL-C gene score genotyping and calculation.

SNP ID	Nearest gene	Risk Allele	Beta coefficient (mmol/l)
rs2479409	*PCSK9*	G	0.051978278
rs629301	*CELSR2*	T	0.146108094
rs1367117	*APOB*	A	0.104732351
rs4299376 (rs6544713)	*ABCG8*	T	0.071114559
rs3757354	*MYLIP*	C	0.036979571
rs1800562	*HFE*	G	0.057408844
rs1564348	*SLC22A1*	T	0.01448151
rs4055111 (rs11220462)	*ST3GAL4*	G	0.050426687
rs8017377	*NYNRIN*	A	0.029480217
rs6511720	*LDLR*	G	0.180760279

Table S4.
Summary of the identified *LDLR* mutations and their *in silico* predicted effect, including calculated LDL-C gene scores for the mutations carriers (presented in bold are the gene scores that are above the top decile cutoff for the control population).

Mutation type/Exon	Mutation	Gene Score	PolyPhen	SIFT	Mutation Taster
Missense					
4	c.326G>A (p.C109Y)	1.03	Probably damaging	Not tolerated	Disease Causing
4	2X c.502G>C (p.D168H)	0.91, N/A	Probably damaging	Not tolerated	Disease Causing
4	c.681C>G (p.D227E)	N/A	Probably damaging	Not tolerated	Disease Causing
9	2X c.1196C>A (p.A399D)	1.03 and 1.07	Possibly damaging	Not tolerated	Disease Causing
11	c.1690A>C (p.N564H)[1]	1.17	Probably damaging	Tolerated	Disease Causing
12	c.1823C>T (p.P608L)	1.09	Probably damaging	Not tolerated	Disease Causing
14	c.2054C>T (p.P685L)	0.97	Probably damaging	Not tolerated	Disease Causing
17	c.2479G>A (p.V827I)	0.92	Probably damaging	Not tolerated	Disease Causing
Nonsense					
4	2X c.682G>T (p.E228*)	0.78 and 0.84	NA	NA	Disease Causing
7	c.1048C>T (p.R350*)	1.11	NA	NA	Disease Causing
8	c.1150C>T (p.Q384*)	0.65	NA	NA	Disease Causing
11	c.1685G>A (p.W562*)	0.95	NA	NA	Disease Causing
Indels					
5	c.695-6_698del	1.23	NA	NA	Disease Causing
12	2X c.1776_1778del p.G592del	N/A	NA	NA	Disease Causing
Intronic					
intron14	c.2140+1G>A	0.58	NA	NA	Disease Causing
intron9	c.1359-31_1359-23 delinsCGGCT	0.92	NA	NA	NA
Large rearrangements					
3_8	c.191-?_1186+?dup	1.03	10kb in frame duplication, peptide elongation		
11_12	c.1587-?_1845+?del	N/A	4kb out of frame deletion, truncated protein		
13_15	c.1846-?_2311+?dup	0.92	7kb out of frame duplication, truncated peptide		

N/A - not available

NA - not applicable

1 – carrier of this variant also has a deletion in exon 17 of *LDLR* c.2393_2401del9 (p.L799_V801del))

Table S5.

All *novel functional APOB* variants identified in the FH cases, including *in silico* predictions of their effect and LDL-C gene scores for the corresponding variant carriers. Using *in silico* mutation prediction tools (PolyPhen2, SIFT, Mutation Taster) the variant located in exon 3 of *APOB* (c.148C>T (p.R50W) has been predicted to be pathogenic by all three algorithms. The mutant Tryptophan is bigger than the wild type Arginine and it is predicted to cause a loss of hydrogen bonds in the core of the protein, which may result in an incorrect folding. The variant has been recently shown to co-segregate with the disease (Thomas et al., *Molecular Genetics & Genomic Medicine2013; 1(3) 155–161*). Other variants include c.598G>A (p.A200T), c.1199G>A (p.R400H), and c.G2700G>T (p.Q900H) in both cases the mutant differs in size and hydrophobicity from the wild type residue, which may affect the folding of the protein as well as the hydrophobic interactions within the protein's core. The novel c.10277C>T (p.A3426V) variant is located near to the LDL-receptor-binding site (*13*), and although it has been predicted as benign/tolerated/polymorphism by the *in silico* tools, it may affect the LDL-R/ApoB interaction. The known FH-causing mutation (p.R3527Q), which was found in two patients, is also listed.

Exon	Variant	Gene Score	PolyPhen	SIFT	MutationTaster	ID
3	c.148C>T(p.R50W)	0.83	Probably Damaging	Not Tolerated	Disease Causing	HYP5062228
6	c.598G>A (p.A200T)	0.98	Possibly Damaging	Not Tolerated	Polymorphism	HYP5269576
10	c.1199G>A(p.R400H)	N/A	Benign	Not Tolerated	Polymorphism	HYP5159267
18	c.G2700G>T (p.Q900H)	1.19	Probably Damaging	Not Tolerated	Polymorphism	HYP5358899
26	c.10277C>T (p.A3426V) and<br>c.6639_6641delTGA (p.2213_2214delD)	1.17	Benign<br>NA	Tolerated<br>NA	Polymorphism<br>Disease Causing	HYP5002222
	2 X c.G10580G>A (p.R3527Q)	0.71 and 1.01	Probably Damaging	Not Tolerated	Disease Causing	HYP5062226 and HYP5062216

NA- not applicable.

Table S6.
Top *p* values of the *novel functional* variant association between cases and controls in the Tier 2 candidate genes.

Gene	Variants in cases (n=71)	Variants in controls (n=1,926)	*p* value
KIAA1875	3	13	0.02
NYNRIN	3	18	0.04
CYC1	1	4	0.17
HFE	1	4	0.17
TOP1	1	4	0.17
ZHX3	2	20	0.18
PVRL2	1	7	0.25
ABCG8	1	18	0.50
OSBPL7	1	19	0.52
CELSR2	1	35	0.73
ABCG5	0	6	1
APOC1	0	2	1
APOE	0	2	1
DCPS	0	8	1
EXOSC4	0	8	1
GPAA1	0	21	1
GRINA	0	9	1
KIRREL3	0	9	1
LPA	0	34	1
LPIN3	0	17	1
MAF1	0	5	1
MYLIP	0	8	1
PARP10	0	11	1
PLCG1	0	25	1
PSRC1	0	4	1
SHARPIN	0	4	1
SORT1	0	9	1
SPATC1	0	12	1
ST3GAL4	0	13	1
TOMM40	0	3	1

Table S7.

Gene burden test of *novel functional* variants for genes in loci associated with FH in family linkage studies.

Chromosomal region	Gene name	Number of rare functional variants		p value
		cases (n=71)	controls (n=1926)	
21q22 (*14*)	*KRTAP10-11*	2	2	0.02
	PFKL	2	4	0.03
	DSCR8	1	0	0.05
	KRTAP11-1	1	0	0.05
	ERG	2	8	0.09
	KRTAP19-8	1	1	0.10
	LRRC3	2	10	0.13
	RCAN1	1	2	0.15
	SIM2	1	2	0.15
	SYNJ1	3	24	0.16
	CBR1	2	15	0.22
	COL18A1	3	30	0.24
	ZNF295	2	17	0.26
	C21orf59	1	5	0.27
	C21orf90	1	5	0.27
	ETS2	1	5	0.27
	C21orf56	1	6	0.31
	KRTAP12-4	1	6	0.31
	PKNOX1	1	6	0.31
	PCNT	5	68	0.33
	BACH1	1	7	0.35
	MX1	1	7	0.35
	BRWD1	2	24	0.39
	PRDM15	2	25	0.41
	HLCS	1	9	0.41
	PRMT2	1	9	0.41
	DOPEY2	3	43	0.43
	SUMO3	1	10	0.44
	MX2	1	11	0.47
	TTC3	2	29	0.48
	AIRE	1	13	0.53
	TRPM2	2	35	0.58
	ABCG1	1	16	0.60
	FTCD	1	17	0.62
	ITSN1	1	20	0.67
	LSS	1	20	0.67
	DSCAM	1	21	0.69
	COL6A2	1	23	0.72
	C21orf2	1	24	0.74
	TRAPPC10	1	24	0.74
	TSPEAR	1	24	0.74
	UMODL1	1	30	0.81
	ITGB2	1	33	0.84
	MCM3AP	1	36	0.86
	URB1	1	57	0.95

16q22 (*15*)	*CMTM2*	2	3	0.02
	HSF4	2	6	0.06
	KCTD19	2	7	0.08
	CES4A	2	8	0.09
	CMTM4	1	2	0.15
	TMEM208	1	3	0.19
	CMTM3	1	5	0.27
	DPEP3	1	5	0.27
	TMCO7	1	5	0.27
	PLEKHG4	2	19	0.30
	C16orf48	1	6	0.31
	CDH16	2	20	0.32
	CES3	1	11	0.47
	CDH3	1	13	0.53
	COG4	1	14	0.55
	GFOD2	1	16	0.60
	TSNAXIP1	1	17	0.62
	FHOD1	1	19	0.65
	SLC12A4	1	19	0.65
	FUK	1	23	0.72
8q24 (*16*)	*WISP1*	3	25	0.17
	ST3GAL1	1	8	0.38
	ZFAT	2	28	0.46
3q25 (*14*)	*HPS3*	3	15	0.06
	ZBBX	2	8	0.09
	NMD3	1	1	0.10
	TRIM59	1	1	0.10
	MLF1	2	11	0.14
	ANKUB1	1	2	0.15
	IL12A	1	2	0.15
	OTOL1	2	13	0.18
	C3orf80	1	3	0.19
	WWTR1	1	4	0.23
	SLITRK3	2	17	0.26
	SMC4	1	6	0.31
	B3GALNT1	1	9	0.41
	MFSD1	1	9	0.41
	SI	2	35	0.58
	MED12L	2	36	0.59
	MECOM	1	24	0.74
	IGSF10	1	34	0.84

Table S8.
All top genes showing a significant excess of *novel functional* variants in cases vs. controls before adjusting for false positive calls. The list includes genes located on chromosome X.

Gene	Number of variants in cases (n=71)	Number of variants in controls (n=1,926)	p value
TMPRSS13	6	7	$2.79x10^{-6}$
CH25H	3	2	$4.3x10^{-4}$
ARMCX2	3	3	$8.29x10^{-4}$
HSPB7	2	0	$1.26x10^{-3}$
KLRC1	2	0	$1.26x10^{-3}$
ZNF645	2	0	$1.26x10^{-3}$
MOAP1	3	4	$1.41x10^{-3}$
RBM25	3	4	$1.41x10^{-3}$
TTC39A	4	13	$2.62x10^{-3}$
ZNF785	4	13	$2.62x10^{-3}$
FZD9	3	6	$3.21x10^{-3}$
HEMK1	3	6	$3.21x10^{-3}$
PCGF3	4	14	$3.28x10^{-3}$
KIF1B	5	24	$3.3x10^{-3}$
ANP32E	2	1	$3.7x10^{-3}$
CABP5	2	1	$3.7x10^{-3}$
CELA2B	2	1	$3.7x10^{-3}$
INSIG2	2	1	$3.7x10^{-3}$
KCTD7	2	1	$3.7x10^{-3}$
MRO	2	1	$3.7x10^{-3}$
NR2E1	2	1	$3.7x10^{-3}$
NXT2	2	1	$3.7x10^{-3}$
PABPC1	2	1	$3.7x10^{-3}$
PODXL	2	1	$3.7x10^{-3}$
PUS3	2	1	$3.7x10^{-3}$
TXNDC15	2	1	$3.7x10^{-3}$
WDR89	2	1	$3.7x10^{-3}$
ZNF720	2	1	$3.7x10^{-3}$

REFERENCE

1. S. A. Miller, D. D. Dykes, H. F. Polesky, A simple salting out procedure for extracting DNA from human nucleated cells. *Nucleic Acids Res* **16**, 1215 (Feb 11, 1988).
2. S. W. Fouchier, J. C. Defesche, M. W. Umans-Eckenhausen, J. P. Kastelein, The molecular basis of familial hypercholesterolemia in The Netherlands. *Human genetics* **109**, 602 (Dec, 2001).
3. A. J. Hooper *et al.*, Genetic analysis of familial hypercholesterolaemia in Western Australia. *Atherosclerosis* **224**, 430 (Oct, 2012).
4. S. Bertolini *et al.*, Spectrum of mutations and phenotypic expression in patients with autosomal dominant hypercholesterolemia identified in Italy. *Atherosclerosis* **227**, 342 (Apr, 2013).
5. C. A. Graham *et al.*, Mutation screening and genotype:phenotype correlation in familial hypercholesterolaemia. *Atherosclerosis* **147**, 309 (Dec, 1999).
6. M. Futema *et al.*, Analysis of the frequency and spectrum of mutations recognised to cause familial hypercholesterolaemia in routine clinical practice in a UK specialist hospital lipid clinic. *Atherosclerosis*, (Apr, 2013).
7. A. Taylor *et al.*, Mutation detection rate and spectrum in familial hypercholesterolaemia patients in the UK pilot cascade project. *Clinical genetics* **77**, 572 (Jun, 2010).
8. R. A. Whittall *et al.*, Development of a high-resolution melting method for mutation detection in familial hypercholesterolaemia patients. *Ann Clin Biochem* **47**, 44 (Jan, 2010).
9. C. D. Mamotte, F. M. van Bockxmeer, A robust strategy for screening and confirmation of familial defective apolipoprotein B-100. *Clinical chemistry* **39**, 118 (Jan, 1993).
10. S. E. Humphries *et al.*, Genetic causes of familial hypercholesterolaemia in patients in the UK: relation to plasma lipid levels and coronary heart disease risk. *J Med Genet* **43**, 943 (Dec, 2006).
11. A. Taylor *et al.*, Multiplex ligation-dependent probe amplification analysis to screen for deletions and duplications of the LDLR gene in patients with familial hypercholesterolaemia. *Clin Genet* **76**, 69 (Jul, 2009).
12. T. M. Teslovich *et al.*, Biological, clinical and population relevance of 95 loci for blood lipids. *Nature* **466**, 707 (Aug 5, 2010).
13. J. Boren *et al.*, Identification of the low density lipoprotein receptor-binding site in apolipoprotein B100 and the modulation of its binding activity by the carboxyl terminus in familial defective apo-B100. *The Journal of clinical investigation* **101**, 1084 (Mar 1, 1998).
14. X. Wang *et al.*, Genome-wide linkage scan of a pedigree with familial hypercholesterolemia suggests susceptibility loci on chromosomes 3q25-26 and 21q22. *PloS one* **6**, e24838 (2011).
15. A. Marques-Pinheiro *et al.*, A fourth locus for autosomal dominant hypercholesterolemia maps at 16q22.1. *European journal of human genetics : EJHG* **18**, 1236 (Nov, 2010).
16. A. Cenarro *et al.*, A presumptive new locus for autosomal dominant hypercholesterolemia mapping to 8q24.22. *Clin Genet* **79**, 475 (May, 2011).
